# Elevated O-GlcNAcylation promotes gastric cancer cells proliferation by modulating cell cycle related proteins and ERK 1/2 signaling

**DOI:** 10.18632/oncotarget.11359

**Published:** 2016-08-17

**Authors:** Mingzuo Jiang, Zhaoyan Qiu, Song Zhang, Xing Fan, Xiqiang Cai, Bing Xu, Xiaowei Li, Jinfeng Zhou, Xiangyuan Zhang, Yi Chu, Weijie Wang, Jie Liang, Tamas Horvath, Xiaoyong Yang, Kaichun Wu, Yongzhan Nie, Daiming Fan

**Affiliations:** ^1^ State Key Laboratory of Cancer Biology and Institute of Digestive Diseases, Xijing Hospital, The Fourth Military Medical University, Xi'an, China; ^2^ Institute of Plastic Surgery of The Chinese PLA, The Fourth Military Medical University, Xi'an, China; ^3^ Department of General Surgery, The General Hospital of People's Liberation Army, 301 Hospital, Beijing, China; ^4^ Department of Molecular Cellular and Developmental Biology, Yale University, New Haven, USA

**Keywords:** O-GlcNAc, gastric cancer, cell cycle, ERK 1/2, clinicopathological parameters

## Abstract

O-GlcNAc transferase (OGT) is the only enzyme in mammals that catalyzes the attachment of β-D-N-acetylglucosamine (GlcNAc) to serine or threonine residues of target proteins. Hyper-O-GlcNAcylation is becoming increasingly realized as a general feature of cancer and contributes to rapid proliferation of cancer cells. In this study, we demonstrated that O-GlcNAc and OGT levels were increased in all six gastric cancer (GC) cell lines as compared with immortal gastric epithelial cells. Downregulation of the O-GlcNAcylation level by silencing OGT inhibited cell viability and growth rate via the cdk-2, cyclin D1 and ERK 1/2 pathways. *In vivo* xenograft assays also demonstrated that the hyper-O-GlcNAc level markedly promoted the proliferation of tumors. Moreover, compared with noncancerous tissues, the O-GlcNAcylation level was increased in cancerous tissues. GC patients with higher levels of O-GlcNAcylation exhibited large tumor sizes (≥5 cm), deep tumor invasion (T3 and T4), high AJCC stages (stage III and IV), more lymph node metastases and lower overall survival. Notably, the phosphorylation level of ERK 1/2 was increased progressively with the increase of O-GlcNAcylation in both SGC 7901 and AGS cells. Consistently, human GC tissue arrays also revealed that ERK 1/2 signaling was positively correlated to O-GlcNAcylation (*r* = 0.348; *P* = 0.015). Taken together, here we reported that hyper-O-GlcNAcylation significantly promotes GC cells proliferation by modulating cell cycle related proteins and ERK 1/2 signaling, suggesting that inhibition of OGT may be a potential novel therapeutic target of GC.

## INTRODUCTION

Gastric cancer (GC) is the third most common cancer in China [1]. Established cell lines and animal models have been vividly intimated the important aspects of GC and provided significant clues to the critical molecular events during the process of the GC development. However, the mechanism behind GC development is still largely unknown.

O-GlcNAcylation is known as a reversible post-translational modification (PTM) regulated by O-GlcNAc transferase (OGT) and O-GlcNAcase (OGA) enzymes [2–6]. One of the remarkable features of cancer cells is the increased in aerobic glycolysis and increased in the rate of glucose uptake and utilization, which is termed the Warburg effect [7]. UDP-GlcNAc, a product of the hexosamine biosynthetic pathway (HBP), is the activated form of GlcNAc and substrate of OGT. A high rate of aerobic glycolysis and glucose uptake would increase the HBP flux, ultimately leading to the elevation of O-GlcNAcylation in cancerous tissues [6–8]. Extensively investigations had confirmed that elevated protein O-GlcNAcylation and changes of OGT and/or OGA expression has a clear functional role in many types of malignancies, including bladder cancer [9], lung cancer [10], prostate cancer [11, 12], hepatocellular carcinoma [13] and colon cancers [14, 15]. O-GlcNAcylation regulates the activities of a wide panel of proteins involved in almost all aspects of cell biology, such as cell proliferation, survival, energy metabolism, migration and invasion [5, 6, 16, 17].

Previous studies revealed that hyper O-GlcNAcylation is associated with the development and progression of GC [18–20]. Recent studies have shown that O-GlcNAc expression levels were progressively increased during the carcinogenesis and progression of GC. Meanwhile, the expression of O-GlcNAcylation in H. pylori-infected chronic gastritis were higher than that in chronic gastritis without H. pylori infection [18]. Yet, the potential effect and mechanism of O-GlcNAcylation in GC are still unclear. Moreover, no evidence is available on whether O-GlcNAcylation can be a hallmark of GC. In this study, we investigated the correlation between the O-GlcNAcylation level and clinicopathological parameters of GC patients. Importantly, we also elucidated the potential role of O-GlcNAcylation in GC proliferation by regulating cell cycle related proteins and activating the extracellular signal regulated kinase (ERK) signaling, which may provide a new sight for the diagnostic and therapeutic strategies of GC in human.

## RESULTS

### O-GlcNAcylation levels are upregulated in gastric cancer (GC)

**Figure 1:**
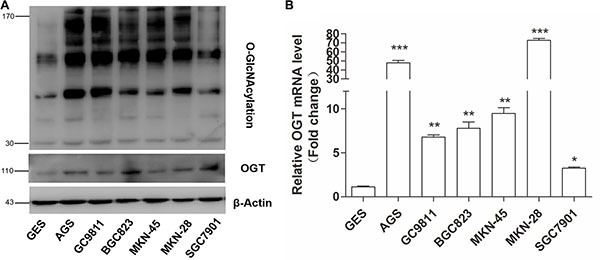
Gastric cancer cells exhibit elevated O-GlcNAcylation and OGT levels (**A**) Western blotting for O-GlcNAcylation and OGT in six gastric cancer cell lines and normal gastric epithelial cells (GES). β-actin was used as a loading control. (**B**) Total RNA was harvested from six gastric cancer cell lines and GES cells, and the levels of OGT mRNA were quantified by qRT–PCR and were normalized to those of β-actin. Normalized OGT mRNA levels were presented relative to GES. Values represent means ± SEM. * represents Student's *t-test* *P < 0.05, **P < 0.01 and ***P < 0.001.

To evaluate the relationship between O-GlcNAcylation and gastric cancer, the OGT and O-GlcNAcylation levels were analyzed in various gastric cancer cell lines and immortal gastric epithelial cells (GES). The results showed that the OGT and O-GlcNAcylation levels were increased in all six gastric cancer cell lines compared with those in GES cells (Figure 1A). Consistently, the OGT mRNA level in all six GC cell lines was increased (Figure 1B). In addition, we found that OGA levels slightly increased in several GC cell lines as compared with GES cells (Supplementary Figure S1). The increase of OGA in GC may be a kind of compensatory change which attempt to “normalize” changing O-GlcNAc levels.

### O-GlcNAcylation promotes the proliferation of gastric cancer cells

**Figure 2:**
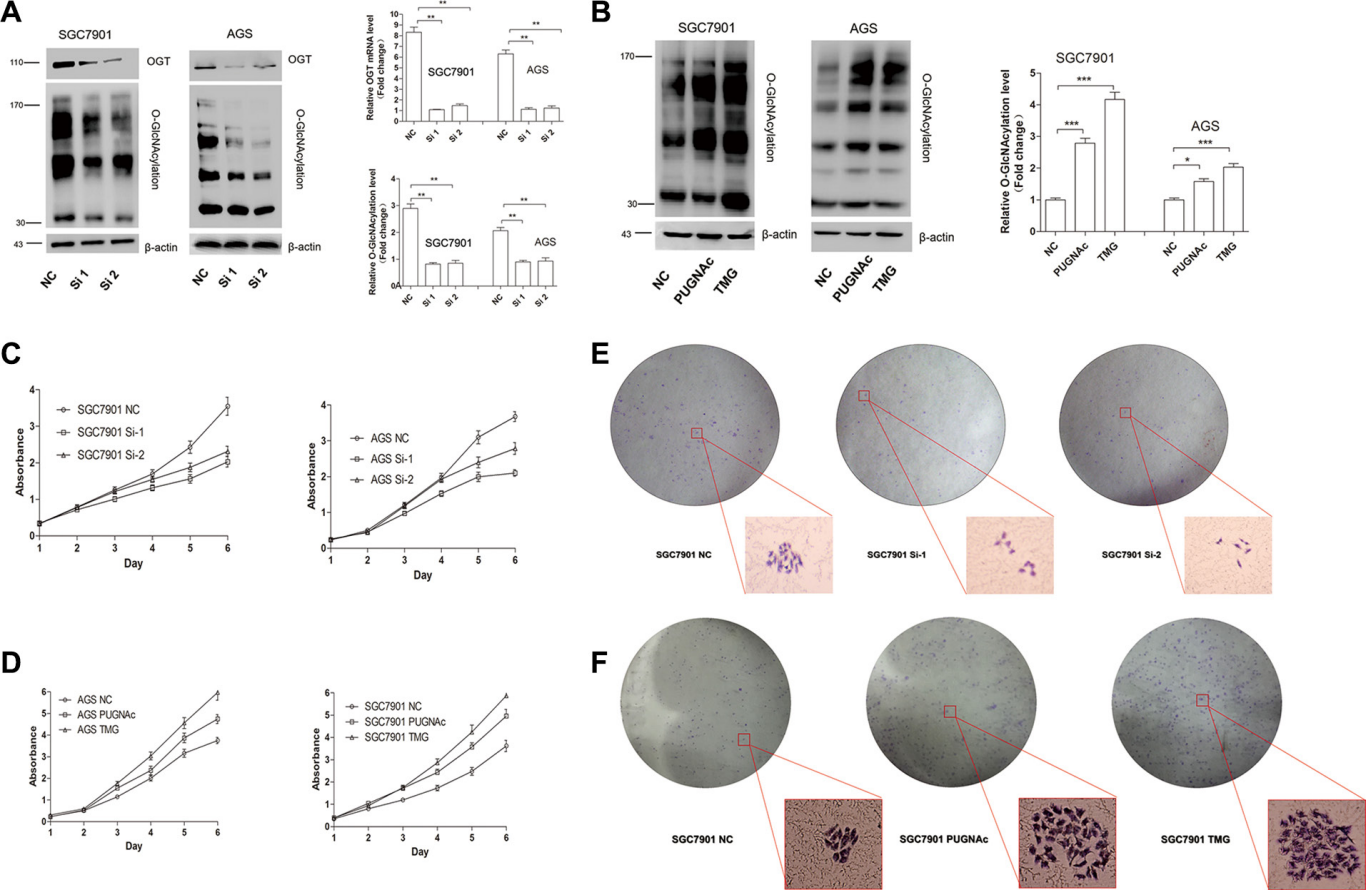
O-GlcNAcylation is associated with gastric cancer cell proliferation *in vitro* (**A**) The expression of OGT and total O-GlcNAcylation level of SGC 7901/AGS cells 72 hours after transfection with NC (negative control), OGT siRNA 1 (Si-1) and Si-2 were determined by an immunoblotting assay. β-actin was used as a loading control. The mean level of OGT or O-GlcNAcylation from three independent experiments is shown on the right. The values shown are expressed as the means ± SEM. (**B**) The total O-GlcNAcylation expression level of SGC 7901/AGS cells after 12 hours of treatment with PUGNAc (100 μmol/L), Thiamet-G (10 μmol/L) or isometric DMSO (negative control, NC) were determined by an immunoblotting assay. β-actin was used as a loading control. The mean level of O-GlcNAcylation from three independent experiments is shown on the right. The values shown are expressed as the means ± SEM. (**C**, **D**) The effect of O-GlcNAcylation on the proliferation rate of SGC 7901/AGS cell lines. The effect on cell proliferation was assessed over 6 days using the cell counting kit-8 assay. The OD450 value is expressed as the mean ± standard deviation. * represents Student's *t-test* *P < 0.05, **P < 0.01 and ***P < 0.001. (**E**, **F**) Effect of O-GlcNAcylation on the clone formation ability of the SGC 7901 cell line. One thousand cells were incubated in 6-well plates for 14 days.

To investigate the function of O-GlcNAcylation in gastric cancer, we used siRNA to knock down endogenous OGT expression in SGC 7901 and AGS cell lines. The efficiency of siRNA silencing was confirmed by Quantitative Real-Time PCR (RT-) and Western blotting (Figure 2A). As expected, silencing OGT led to a significant reduction in overall O-GlcNAcylation compared with control cells (Figure 2A). In addition, to prove that an elevated overall O-GlcNAcylation level in SGC 7901 and AGS cell lines can stimulate cell proliferation, the OGA inhibitors PUGNAc and Thiamet-G (TMG) were used. The drug efficiency was confirmed by Western blotting (Figure 2B).

Next, cell viability was measured by the CCK-8 assay for 6 days. Compared with scrambled negative control siRNA, OGT siRNA significantly inhibited cell proliferation (Figure 2C, *P < 0.01*). By contrast, the upregulation of O-GlcNAcylation caused by the OGA inhibitors PUGNAc and Thiamet-G (TMG) enhanced cell proliferation (Figure 2D, *P < 0.05*). Meanwhile, a clone formation assay of SGC 7901 cells showed similar results (Figure 2E). These results suggest that O-GlcNAcylation can promote gastric cell proliferation.

### O-GlcNAcylation can regulate cell cycle progression

**Figure 3:**
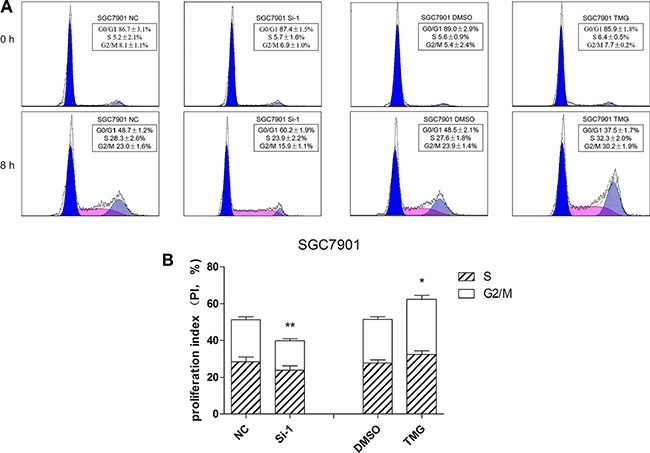
O-GlcNAcylation is involved in the regulation of cell cycle progression (**A**) Cell cycle analysis of SGC 7901 cells after 12 hours of Thiamet-G (TMG, 10 μmol/L) treatment or after 48 hours of transfected OGT siRNA. All of the cells were synchronized in the G0/G1 phase by serum starvation for 24 hours and were collected to analyze the cell cycle by flow cytometry after 8 hours of serum stimulation. (**B**) The proliferation index is expressed as the mean ± SD. *P < 0.05 and **P < 0.01 vs. the negative control.

Reduced growth can be attributed to cell cycle defects. Thus, we wondered whether O-GlcNAcylation could modulate cell cycle progression. SGC 7901 cells were prepared for flow cytometry analysis after Thiamet-G (TMG, 10 μmol/L) treatment or after OGT siRNA transfection. We synchronized all of the cells in G0 phase by 24-hour serum starvation. The cells were then collected, stained with propidium iodide (PI) and analyzed by flow cytometry after 8 hours of serum stimulation (Figure 3A). As a result, the reduction of OGT in SGC 7901 cells caused a significant accumulation of cells in the G1 phase compared with control siRNA-infected cells: 60.2 ± 1.9% G1 content in OGT siRNA-infected cells relative to 48.7 ±1.2% in control siRNA cells (Figure 3B). Accordingly, the upregulation of the O-GlcNAcylation level by TMG led to increased cell proportion in the S- and G2/M-phases compared with control (Figure 3B).

### The downregulation of O-GlcNAc level blocks the ERK 1/2 pathway and decreases cdk2 and cyclin D1 *in vitro*

**Figure 4:**
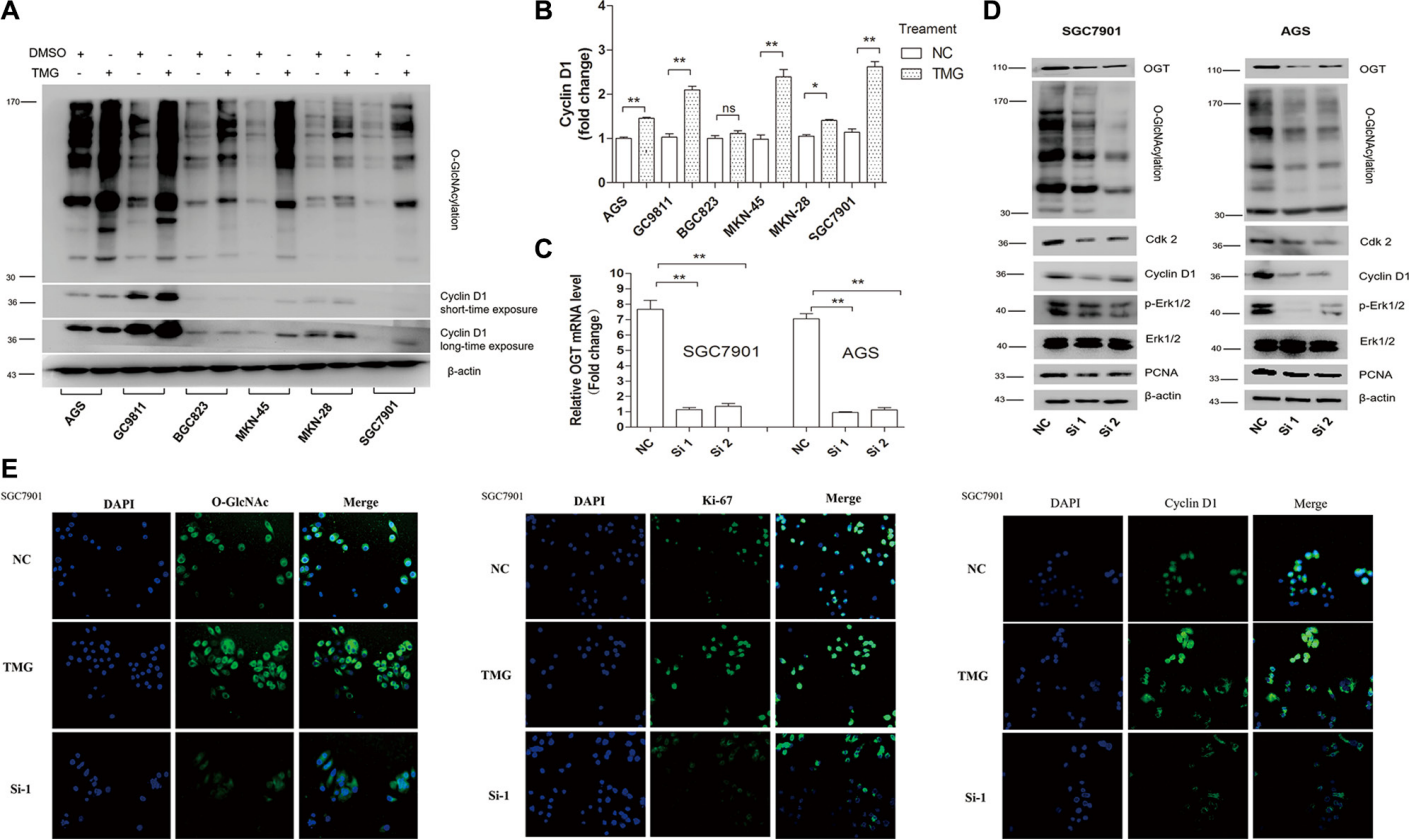
Silencing of OGT inhibits GC cell proliferation by blocking the ERK pathway and down-regulating Cdk2 and Cyclin D1 (**A**, **B**) The expression of the Cyclin D1 level in six GC cell lines after 12 hours of treatment with Thiamet-G (10 μmol/L) or isometric DMSO (NC) were determined by immunoblotting assay. β-actin was used as a loading control. The mean level of OGT or O-GlcNAcylation from three independent experiments is shown on the right. The values shown are expressed as the means ± SEM. *P < 0.05 and **P < 0.01 vs. negative control. ns indicates no significance. (**C**, **D**) Down-regulated expression of OGT in SGC 7901 and AGS resulted in the downregulated expression of Cdk-2, Cyclin D1, p-Erk and PCNA. The efficiency of interference is shown on the left. The values shown are expressed as the means ± SEM. *P < 0.05 and **P < 0.01 vs. negative control. (**E**) Immunofluorescence analysis of the O-GlcNAcylation level (green), Ki-67 (green) and Cyclin D1 (green) in SGC 7901 treated with TMG or silenced OGT. Nuclei were stained with 4′,6-diamidino-2-phenylindole (DAPI; blue).

Next, we explored the mechanism by which O-GlcNAcylation could regulate GC cell proliferation. Cyclin D1 is the key molecule regulating the G0/G1 transition. The levels of O-GlcNAcylation and cyclin D1 in 6 GC cell lines after 12 hours of treatment with Thiamet-G (10 μmol/L) or isometric DMSO were detected by immunoblotting assay. The results showed that O-GlcNAcylation upregulation by Thiamet-G could increase cyclin D1 levels in 5 GC cell lines (Figure 4A and 4B). We then selected SGC 7901 and AGS for further investigation. The immunoblotting assay showed that the expression levels of cdk-2 and cyclin D1, proteins involved in the regulation of the mitotic cell cycle, were decreased when the OGT gene was downregulated by siRNA (Figure 4C and 4D). Abnormal activation of extracellular signal-regulated kinase (ERK) signaling pathway leads to inappropriate cell proliferation and survival. In our studies, we found that the phosphorylation level of ERK1/2 was significantly decreased by OGT siRNA, indicating that the O-GlcNAcylation level may regulate GC cell proliferation through the ER signaling pathway (Figure 4D). Immunofluorescence analysis of O-GlcNAc, Ki-67 and cyclin D1 further validated that O-GlcNAcylation could promote GC cell proliferation (Figure 4E)

### An increased O-GlcNAc level promotes gastric tumor growth *in vivo*

**Figure 5:**
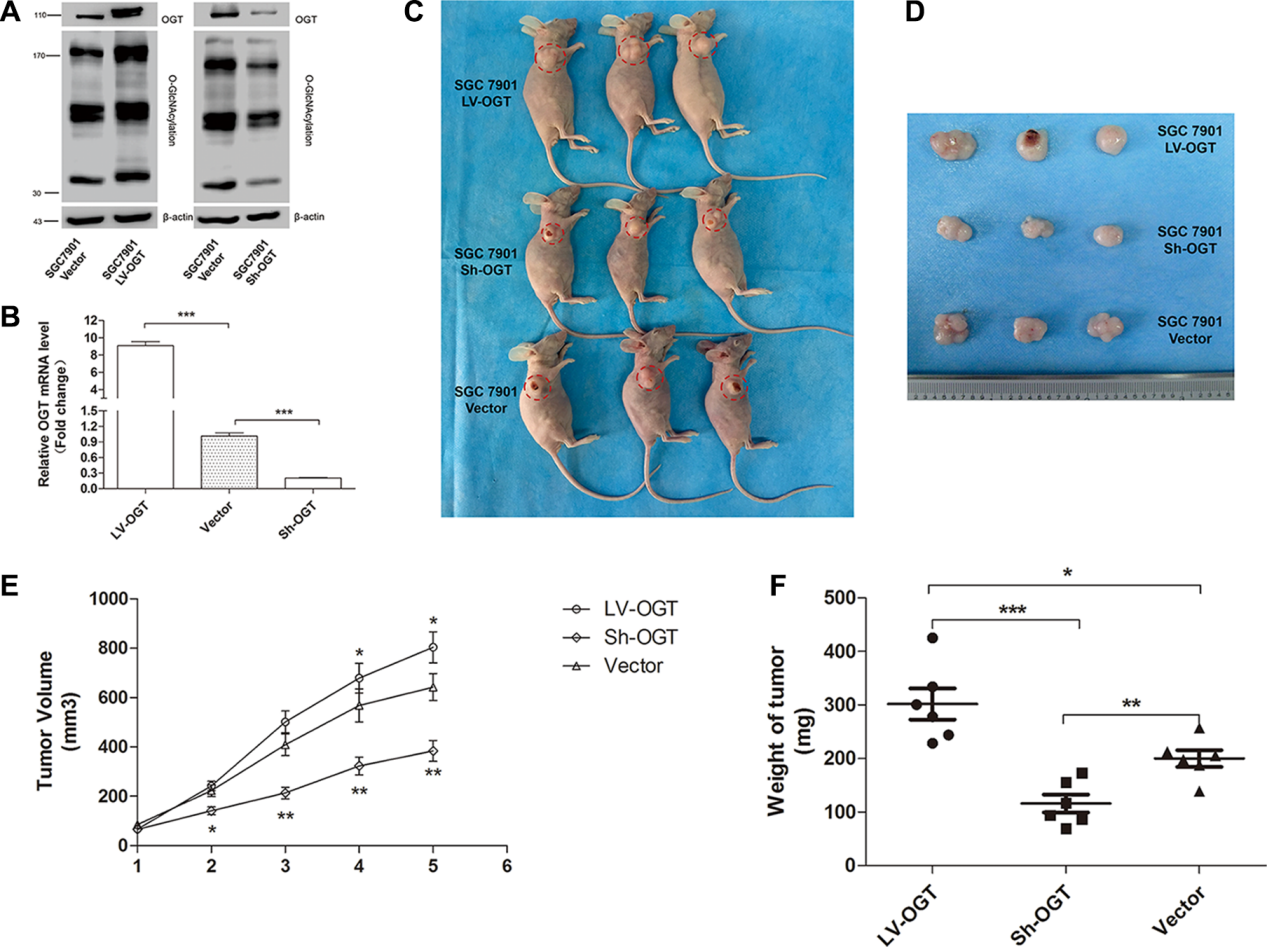
Effect of O-GlcNAcylation on gastric tumor growth in a xenograft model (**A**) The expression of OGT and total O-GlcNAcylation levels of SGC 7901 LV-OGT, Sh-OGT and Vector cells were determined by an immunoblotting assay. β-actin was used as a loading control. (**B**) The mean mRNA level of the OGT gene in SGC 7901 LV-OGT, Sh-OGT and Vector cell lines from three independent experiments is shown. The values shown are expressed as the means ± SEM. ***p < 0.001 (**C**) The mice were sacrificed five weeks after injection, and representative anatomical photos of mice injected with the stable transfectants of SGC 7901 LV-OGT, Sh-OGT or Vector cell lines are shown. (**D**) Representative anatomical photos of tumors from mice are presented. (**E**) Tumor volumes were measured weekly, and the mean volume of each group was calculated. (**F**) Tumor weights were examined immediately after the mice were sacrificed, and statistical analysis was performed. Data are expressed as the means ± SEM (*n* = 6). *p < 0.05, **p < 0.01.

SGC 7901 cells were infected with recombinant lentivirus expressing OGT (LV-OGT), small hairpin RNA (Sh-OGT) or a vector control lentivirus (Vector), and stable clones were established (named SGC 7901 LV-OGT, SGC 7901 Sh-OGT and SGC 7901 Vector, respectively). The upregulation or downregulation of OGT expression was confirmed by qPCR and Western blot analyses (Figure 5A and 5B). To further explore the role of O-GlcNAcylation, we inoculated equal amounts of SGC 7901 LV-OGT, SGC 7901 Sh-OGT and SGC 7901 Vector cells into the front flank of female athymic nude mice. Compared with the SGC 7901 Vector groups, the size and weight of the tumors were significantly lower in the SGC 7901 Sh-OGT group (*P* < 0.01, Figure 5). Additionally, the size and weight of the SGC 7901 LV-OGT group were the highest (*P* < 0.05, Figure 5).

### Clinicopathological significance of O-GlcNAcylation in GC patients

**Figure 6:**
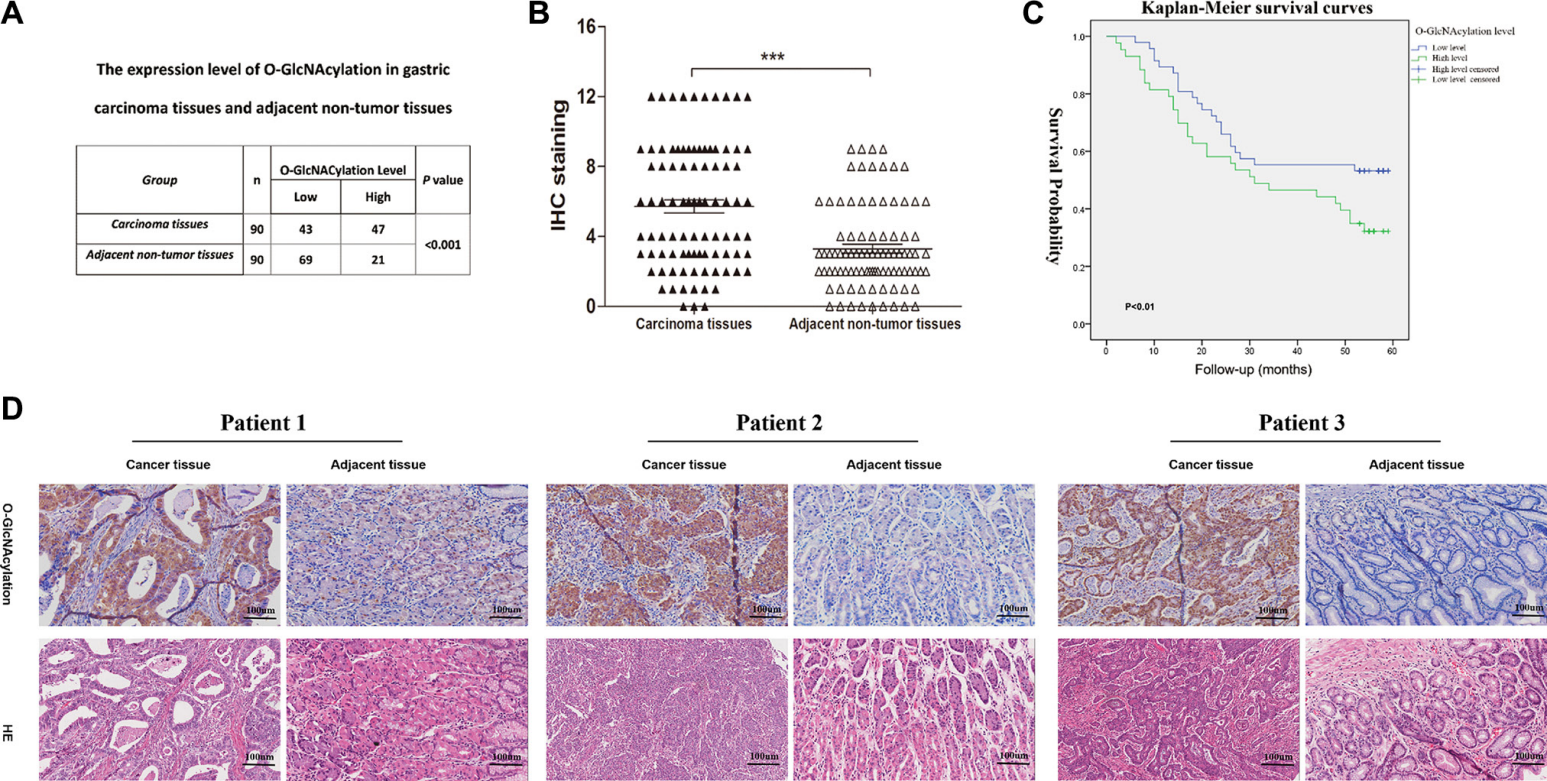
The O-GlcNAcylation levels are positively correlated with the malignancy of gastric cancer (**A**) The O-GlcNAcylation level in 90 cases of cancerous tissues and matched normal counterparts. (**B**) The O-GlcNAcylation levels were scored with semiquantitative immunohistochemical (IHC) analysis. O-GlcNAcylation was upregulated in GC tissues compared with matched normal counterparts (*n* = 90). *** represents Student's *t-test P* < 0.001. (**C**) Kaplan-Meier curves depicted the overall survival of patients with GCs (*n* = 90). The curves were stratified based on the O-GlcNAcylation level. Overall survival was defined as the interval between the date of surgery and date of death or last follow-up. (**D**) Immunohistochemical staining for O-GlcNAcylation in GC tissues and matched normal counterparts from three patients.

To characterize the clinicopathological relevance of O-GlcNAcylation in GC patients, we analyzed the relationships between the O-GlcNAcylation and clinicopathological parameters. Compared with noncancerous tissues in 90 cases of gastric cancer patients, the O-GlcNAcylation level was increased in cancerous tissues (Figure 6A and 6B). Then cancerous tissues and matched normal counterparts were divided into the high O-GlcNAcylation group (score: 5–12) and low O-GlcNAcylation group (score: 0–4), respectively (Figure 6B). High expression of O-GlcNAcylation was detected in 47 (52.22%) cases of GC tissues and in 21 (23.33%) of the matched normal counterparts (*P* < 0.01, Figure 6A). Consistent with other findings [11, 21], the overall survival in patients with a high O-GlcNAcylation level was remarkably reduced (Figure 6C, *P* < 0.01). Meanwhile, the tumor size, invasion depth, lymph node metastases and AJCC stage also affected the overall survival of GC patients as assessed by univariate analysis (Table 1, *P* < 0.05). In addition, GC patients with a large tumor size (≥ 5 cm), deep tumor invasion (T3 and T4), high AJCC stage (stage III and IV), lymph node metastases, or lymphatic and/or vascular invasion had significantly higher levels of O-GlcNAcylation (Table 2, *P* < 0.05). Multivariate analyses also revealed that a high O-GlcNAcylation level was an independent risk factor for the poor prognosis of GC (Table 3, *P* < 0.05).

**Table 1:** Prognostic factors in patients with gastric cancer by univariate analysis

Parameter	*n*	Hazard Ratio	95% Confidence Interval	*P* value
**Gender**				
Male	62	1.16	0.65–2.08	0.61
Female	28			
**Age**				
< 60	35	1.37	0.76–2.44	0.29
≥ 60	55			
**Location**				
Cardia	8			0.45
Fundus/body of stomach	29	0.33	0.04–2.78	0.31
Antrum	50	0.46	0.06–3.45	0.45
Diffuse	3	0.62	0.08–4.55	0.64
**Tumor size**				
< 5 cm	37	2.04	1.14–3.70	0.02
≥ 5 cm	53			
**Invasion depth**				
Without Infiltration into Serous layer	15	3.33	1.20–9.009	0.02
Infiltration into serous layer	75			
**Lymph node metastasis**				
Negative	21	5.56	1.96–14.28	< 0.01
Positive	69			
**Lymphatic and/or vascular invasion**				
Negative	66	1.56	0.71–3.45	0.26
Positive	24			
**AJCC stage**				
I/II	34	2.70	1.39–5.26	< 0.01
III/IV	56			
**O-GlcNAcylation level**				
High	47	2.27	1.28–4.00	< 0.01
low	43			

**Table 2:** Association of O-GlcNAcylation level with clinicopathological parameters of patients with gastric cancer

Parameter	*n*	O-GlcNACylation Level		*P* value
		Low	High	
**Gender**				
Male	62	31	31	0.53
Female	28	12	16	
**Age**				
< 60	35	19	16	0.93
≥ 60	55	24	21	
**Location**				
Cardia	8	2	6	0.52
Fundus/body of stomach	29	14	15	
Antrum	50	26	24	
Diffuse	3	1	2	
**Tumor size**				
< 5 cm	37	28	9	**< 0.001**
≥ 5 cm	53	15	38	
**Invasion depth**				
Without Infiltration into Serous layer	15	11	4	**0.03**
Infiltration into serous layer	75	32	43	
**Lymph node metastasis**				
Negative	21	16	5	**< 0.01**
Positive	69	27	42	
**Lymphatic and/or vascular invasion**				
Negative	66	36	30	**0.03**
Positive	24	7	17	
**AJCC stage**				
I/II	34	20	14	**0.02**
III/IV	56	23	33	

**Table 3:** Multivariate analysis using the Cox proportional hazards model

Parameter	*n*	Hazard Ratio	95% Confidence Interval	*P* value
**Tumor size**				
< 5 cm	37	1.52	0.81 – 2.86	0.189
≥ 5 cm	53			
**Invasion depth**				
Without Infiltration into Serous layer	15	1.46	0.45 – 4.69	0.524
Infiltration into serous layer	75			
**Lymph node metastasis**				
Negative	21	3.45	1.01 – 11.76	**0.048**
Positive	69			
**Lymphatic and/or vascular invasion**				
Negative	66	1.26	0.69 – 2.33	0.455
Positive	24			
**AJCC stage**				
I/II	34	1.151	0.38 – 1.97	0.737
III/IV	56			
**O-GlcNAcylation level**				
High	47	1.99	1.07 – 3.72	**0.031**
low	43			

### Correlation of p-ERK 1/2 with O-GlcNAcylation *in vitro* and *in vivo*

**Figure 7:**
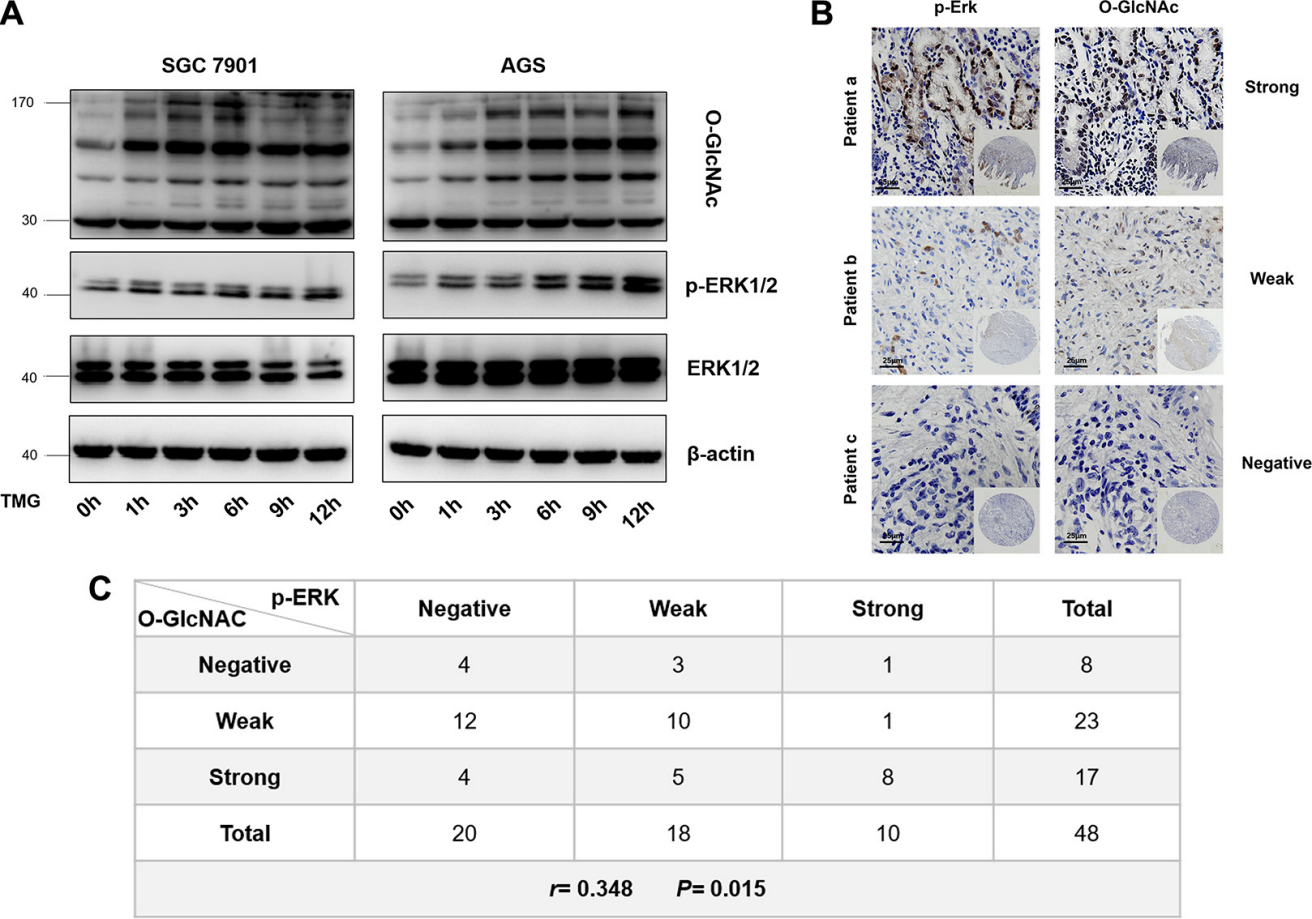
Correlation of p-ERK 1/2 with O-GlcNAcylation *in vitro* and *in vivo* (**A**) The level of p-ERK 1/2 in SGC 7901 and AGS after treatment with TMG (10 μmol/L) at different times (0 h, 1 h, 3 h, 6 h, 9 h or 12 h). (**B**) Immunohistochemical staining for p-ERK 1/2 and O-GlcNAc in GC tissue from the same patients. (**C**) Pearson correlation analysis of p-ERK 1/2 and O-GlcNAcylation in GC tissues.

To further investigate the correlation between p-ERK 1/2 and O-GlcNAcylation, we detected the level of p-ERK 1/2 in SGC 7901 and AGS cells after treatment with TMG (10 μmol/L) at different times (0 h, 1 h, 3 h, 6 h, 9 h or 12 h). The results showed that the level of p-ERK 1/2 was also increased progressively with the increase in O-GlcNAcylation in both SGC 7901 and AGS cells (Figure 7A). To further verify the correlation between the levels of p-ERK 1/2 and O-GlcNAcylation *in vivo*, we analyzed the levels of p-ERK 1/2 and O-GlcNAcylation in two identical gastric cancer TMAs (provided by Xijing Hospital of Digestive Diseases, Fourth Military Medical University), which were constructed with formalin-fixed paraffin-embedded gastric neoplastic tissues of 48 gastric cancer patients. We divided the levels of p-ERK 1/2 and O-GlcNAcylation into three groups (Figure 7B). The results showed that there was a positive correlation between the levels of p-ERK 1/2 and O-GlcNAcylation *in vivo* by Pearson correlation analysis. (*r* = 0.348; *P* = 0.015) (Figure 7C).

## DISCUSSION

Studies in recent years have shown that a wide panel of oncogenic factors can be modified by O-GlcNAc and up-regulation of O-GlcNAcylation is believed to participate in tumor malignancy [6]. Such as the modification of p53 and c-Myc with O-GlcNAc would increase their stabilities by blocking ubiquitin degradation [12, 22]. In addition to stabilizing oncogenic factors, O-GlcNAcylation could regulate their transcriptional activities. For example, the O-GlcNAcylation of p65 at Thr322 and Thr352 is critical for p65 promoter binding [14]. Furthermore, the OGT and O-GlcNAcylation levels were high in late S phase and peaked at the M phase, indicating it may participate in the regulation of cell cycle progression [22, 23]. In our study, we found that the upregulation of O-GlcNAcylation promoted cell proliferation and depletion of OGT suppressed cell proliferation, which were accompanied by consistent alterations of the cell cycle and cell cycle-related proteins such as cdk-2, cyclin D1 and ERK 1/2. All of these results point to the ability of O-GlcNAcylation to enhance proliferation by modulating cell cycle progression and the ERK 1/2 pathway.

Clinical studies have also found that the high level of O-GlcNAcylation participate in the malignant clinicopathological features of various cancer patients [11, 13]. In breast ductal carcinomas, poorly differentiated tumors (grade II and III) displayed significantly higher *OGT* expression than grade I tumors [24]. Additionally, the *OGT* mRNA level was even higher in the urine of bladder cancer patients with invasive or advanced stages (grade II and III) compared with those with early stages (grade I) [9]. In human laryngeal cancer, the increased level of *OGT* mRNA was also related to a larger tumor size, nodal metastases, and a higher grade as well as the incidence of disease recurrence [25]. An independent cohort of colorectal cancer patients from the Moffitt Cancer Center (GSE17536) [21] also showed that patients with high levels of OGT exhibited worse prognoses. In this study, we explored the clinical significance of the O-GlcNAcylation level in numerous GC tissues and found that its level was positively correlated with a large tumor size (≥ 5 cm), deep tumor invasion (T3 and T4), high AJCC stage (stage III and IV), lymph node metastasis and the overall survival of patients with a high O-GlcNAcylation level was significantly shortened. Meanwhile, we detected the expression of OGT and ERK 1/2 in five cases of gastric cancerous tissues and matched normal counterparts. The result also showed that the level of OGT was increased in cancerous tissues (Supplementary Figure S3).

The extracellular signal-regulated kinases ERK1 and ERK2 (ERK 1/2) cascade was first discovered as a classical signal-transduction pathway of the MAPK family, which regulates various cellular processes in normal and cancer tissues [26, 27]. In our study, we have elucidated that phosphorylated ERK 1/2 was decreased when OGT was silenced in GC cell lines. Similarly, high level of O-GlcNAcylation promoted high glucose-induced cardiac hypertrophy via ERK 1/2 [28], indicating that the regulation of the ERK 1/2 pathway by O-GlcNAcylation plays an important role in many diseases. Importantly, a recent study has revealed that activated ERK 1/2 signaling leads to increased expression of OGT and O-GlcNAcylation levels in various cancers [29], indicating that there is possibly positive feedback between the ERK 1/2 signaling pathway and the O-GlcNAcylation level in cancer cells. Although many colleagues have reported the correlation between O-GlcNAcylation and the ERK 1/2 pathway [28–31], the mechanism underlying the O-GlcNAc regulation of ERK 1/2 activation in gastric cancer is unclear. We predicted that there are O-GlcNAc sites in ERK 2 using the YinOYang 1.2 Server (www.cbs.dtu.dk/services/YinOYang) (Supplementary Figure S2A), which indicates that ERK 1/2 may be directly modified by O-GlcNAc. It is the accepted conclusion that O-GlcNAcylation has extensive crosstalk with phosphorylation to regulate signaling, transcription and the cytoskeleton in response to intracellular or extracellular stress [8, 32]. Thus, we hypothesized that the O-GlcNAcylation of ERK 1/2 can promote its phosphorylation and then activate this pathway, a finding that needs further research to confirm. In addition to direct O-GlcNAc modification, activation of this pathway may occur through the regulation of upstream ERK signaling components such as MEKs. Skorobogatko et al. proved that MEK2, a signaling component of the ERK 1/2 pathway, can be modified by O-GlcNAc and promotes the activation of the ERK 1/2 pathway at neuronal synapses. [33]

There is also a close relationship between O-GlcNAcylation and cell cycle regulation. A high level of glucosamine, which would increase the level of O-GlcNAcylation, could increase cell cycle regulatory proteins, including cyclin D1, CDK4, cyclin E, and CDK2, in mouse embryonic stem cells [34]. In addition, the reduction of O-GlcNAcylation in breast cancer cells leads to the inhibition of tumor growth and is associated with decreased cell cycle progression [35]. Meanwhile, *OGT* interference blocking cyclin D1 expression and the catalytic activity of OGT are necessary for the G0/G1 transition [36]. Similar phenomena were also confirmed in our experiments. Our group has found that the upregulation of O-GlcNAcylation by TMG could increase cyclin D1 in various GC cell lines. Cyclin D1 and cdk-2 were decreased when OGT was silenced in SGC 7901 and AGS cell lines. It is known that O-GlcNAcylation can stabilize β-catenin [37], a transcription factor that can promote cyclin D1 transcription, which may have increased the cyclin D1 level as observed in our study. Interestingly, using the YinOYang 1.2 Server (www.cbs.dtu.dk/services/YinOYang), we predicted that there are two O-GlcNAc sites in cyclin D1 (Supplementary Figure S2B). However, further studies are needed to clarify whether the levels of these proteins are regulated by O-GlcNAcylation through the above-mentioned mechanisms.

In summary, our results have demonstrated that hyper-O-GlcNAc level markedly promoted the proliferation of tumors and indicated poor prognosis of GC patients. In this study, we have proven that O-GlcNAcylation can enhance GC cell proliferation mechanistically by orchestrating the expression of cell cycle-related proteins and ERK 1/2 pathway. And thus we propose that inhibition of hyper-O-GlcNAcylation may as a potential novel therapeutic target for cancer treatment.

## MATERIALS AND METHODS

### Cell culture

GES, GC9811, MKN-45, MKN-28, BGC 823, SGC 7901 and AGS cells were maintained in DMEM medium (Gibco, Grand Island, NY, USA) supplemented with 10% fetal bovine serum (Bioind, Kibbutz Beit Haemek, Israel) and 1% penicillin/streptomycin (Gibco, Grand Island, NY, USA) at 37°C in a humidified incubator with 5% CO_2_.

### PUGNAc or Thiamet-G treatment

PUGNAc (Sigma-Aldrich, St. Louis, USA) was dissolved in dimethyl sulfoxide (Sigma-Aldrich, St. Louis, USA) to a concentration of 200 mmol/L and was diluted to a final concentration of 100 μmol/L in RPMI-1640 medium. Thiamet-G (Calbiochem, San Diego, USA) was dissolved in DMSO to a concentration of 40 mmol/L and was diluted to a final concentration of 10 μmol/L in DMEM medium. Prior to PUGNAc or Thiamet-G treatment, the cells were incubated with DMEM medium containing 10% FBS and 1% penicillin/streptomycin to reach 80% cell confluency. Next, cells were treated with PUGNAc (100 μmol/L) or Thiamet-G (10 μmol/L) in the absence of FBS for 12 h.

### Cell viability assays

Cells were seeded in 96-well plates (1 × 10^4^ cells/well), cultivated at 37°C in a 5% CO_2_ humidified incubator for 24 h. Ten microliters of Cell Counting Kit-8 solution (Dojindo, Kumanoto, Japan) were added to each well and were incubated at 37°C in a 5% CO_2_-humidified incubator for 2 h. Spectrometer Varioskan^®^ Flash (ThermoFisher, Waltham, USA) was used to measure the 450-nm absorbance. A proliferation curve was drawn with the time as the abscissa and average absorbance value in each group as the ordinate. Triplicate reactions were performed for the experiment.

### Colony formation assay

Cells were seeded in 6-well plates (1000 cells/well) and were incubated at 37°C in a 5% CO_2_-humidified incubator. After 2 weeks, the cells were stained with Gentian violet (Beyotime Biotechnology, Shanghai, China). The experiments were repeated three times.

### Immunofluorescence assay

Cell immunofluorescence was performed as previously reported [38].

### Lentivirus infection

Stable transfectant SGC 7901 cell lines overexpressing OGT (SGC 7901 LV-OGT) or with OGT knockdown by small hairpin RNA (SGC 7901 Sh-OGT) were generated by lentiviral transduction using a GV341 vector (GeneChem Co., Ltd, Shanghai, China). An empty vector was used as the negative control (SGC 7901 Vector). Sequences are available from the authors on request.

### Xenografted tumors in nude mice

Eighteen female BALB/c nude mice (5 to 6 weeks old; The Fourth Military Medical University, Xi'an, China) were randomly divided into three groups (LV-OGT, Sh-OGT and Vector groups). All of the mice were maintained under specific pathogen-free conditions. This study was carried out in strict accordance with the recommendations in the Guide for the Care and Use of Laboratory Animals of the National Institutes of Health. All of the procedures in the studies involving animals were performed in accordance with the ethical standards of The Fourth Military Medical University. The cells were cultured, and single-cell suspensions were prepared with cells at the log phase. Cells (1 × 10^7^ cells) were subcutaneously injected into the right side of the back of the nude mice to establish the subcutaneous xenograft tumor model. The mice were checked daily, and the body weight and tumor size were measured every week. After 5 weeks, all of the mice were sacrificed under deep anesthesia. The final volume and weight of each tumor were recorded. The tumor volumes were calculated using the following formula: tumor volume (mm^3^) = [length (mm)×width (mm)^2^]π/6.

### Statistical analysis

We used Prism 5 software (San Diego, CA, USA) and SPSS 18.0 (Chicago, IL, USA) for statistical analysis. All of the values are expressed as the means ± SD. The usefulness of the O-GlcNAc level to predict the risk of GC development was assessed using the Kaplan–Meier and Cox proportional hazards model analyses. The results were compared using Student's *t-test*. A *P value* < 0.05 was considered to indicate a significant difference.

## SUPPLEMENTARY MATERIALS FIGURES AND TABLES


