# Supplementary material for: Elevated O-GlcNAcylation promotes gastric cancer cells proliferation by modulating cell cycle related proteins and ERK 1/2 signaling
Source: Oncotarget. 2016 Aug 17;7(38):61390–402. doi: 10.18632/oncotarget.11359 (PMC5308659; doi:10.18632/oncotarget.11359)
Supplement: Supplementary file 1 [file oncotarget-07-61390-s001.pdf]

Elevated O-GlcNAcylation promotes gastric cancer cells proliferation by modulating cell cycle related proteins and ERK 1/2 signaling

Supplementary Materials

SUPPLEMENTAL EXPERIMENTAL PROCEDURES

Western blot analysis

Cells were washed with PBS three times and collected in RIPA lysis buffer (Beyotime Biotechnology, Shanghai, China) supplemented with protease inhibitor cocktail (Calbiochem, San Diego, USA) and protease inhibitor cocktail (Calbiochem, San Diego, USA). The protein concentration was determined by Coomassie Brilliant Blue (Beyotime Biotechnology, Shanghai, China) staining. A 10% separating gel and a 5% staking gel were prepared. Each well was loaded with 15 ul of sample. After electrophoresis, proteins were transferred to a polyvinylidene difluoride membrane (Merck Millipore, Darmstadt, Germany). After blocking with Tris-buffered saline with 0.1% Tween-20 (TBS-T) containing 5% skim milk for 1 hour at RT, the primary monoclonal antibody was added to the membrane for incubation overnight at 4°C. The next day, membranes were incubated with corresponding secondary antibodies for 1 hour at RT and signals were detected in a BIO-RAD ChemiDoc XRS Imaging system. The ratio of the gray value between target protein and β-actin represented the relative amount of protein.

Cell cycle analysis

All the cells were synchronized in G0/G1 phase by 24 h serum starvation. Then approximately 1×10^6 cells were collected and fixed by 70% ethan.

After 8 h serum stimulation. Cells were stained with propidium iodide (Sigma-Aldrich, st Louis, USA) and analyzed by FACScan flow cytometer (BD Biosciences, Franklin Lakes, NJ). The results were analyzed using Multicycle-DNA Cell Cycle Analyzed Software. The Proliferation index (PI) was calculated as previously described: PI = (S+G2)/(S+G2+G1) [1].

Quantitative real-time polymerase chain reaction (qRT-PCR)

The total RNA was extracted from samples using the TaKaRa MiniBEST Universal RNA Extraction kit (TaKaRa, Tokyo, Japan). The RNA extract was measured using an ultraviolet spectrophotometer at 260 and 280 nm. Only samples with an OD260/OD280 ratio of 1.8 to 2.0 were used for subsequent analysis. Complementary DNA was obtained by reverse transcription according to the manufacturer's instructions (TaKaRa, Tokyo, Japan). The expression of mRNA level was analyzed by Real Time Fluorescent Quantitative PCR Instrument with CFX96 software (Bio-Rad, CA, USA) according to the manufacturer's instructions. The cycling parameters were as follows: 95°C for 30 seconds, followed by 45 cycles of 95°C for 5 seconds and 60°C for 30 seconds. A melting curve analysis was then performed. The relative amount of mRNA were normalized to β-actin.

Short interfering RNA (siRNA) transfection

Double-stranded siRNA targeting human OGT (purchased from Biomics Biotechnologies Co., Ltd, Nantong, China), Opti-MEM (Invitrogen, Karlsruhe, Germany) media and HiPerFect® Transfection Reagent (QIAGEN, Dusseldorf, Germany) were mixed and incubated according to the manufacturer's instructions. In all experiments, scrambled siRNA served as a control. Cells were analyzed 48 h post-transfection.

Tissue microarray

GC tissue microarrays (HStm-Ade180Sur-07 tissue array) were purchased from Outdo Biotech Co.,Itd (Shanghai, China). Each array included 90 cases of gastric malignant tissues and their matched normal counterparts. Another microarray containing 48 GC cases was provided by Xijing Hospital of Digestive Diseases, Fourth Military Medical University.

Immunohistochemistry

Monoclonal antibodies against O-GlcNAc (Cell Signaling, Boston, USA) was evaluated. Tissue staining was performed according to the VECTASTAIN Elite ABC system protocol (Vector Laboratories, PK-6101, USA). Both the immunohistochemical staining score of positive cells and the intensity of the positive cells on each slide were calculated using the semi-quantitative scoring method [2]. Evaluation of immunostaining intensity was performed as previously described [3].

REFERENCES

1. Liang J, Pan Y, Zhang D, Guo C, Shi Y, Wang J, Chen Y, Wang X, Liu J, Guo X, Chen Z, Qiao T, Fan D. Cellular prion protein promotes proliferation and G1/S transition of human gastric cancer cells SGC7901 and AGS. Faseb j. 2007; 21:2247–2256.
2. Robinson AR, Kwek SS, Kenney SC. The B-cell specific transcription factor, Oct-2, promotes Epstein-Barr virus latency by inhibiting the viral immediate-early protein, BZLF1. PLoS pathogens. 2012; 8:e1002516.
3. Zhou L, Shang Y, Liu C, Li J, Hu H, Liang C, Han Y, Zhang W, Liang J, Wu K. Overexpression of PrPc, combined with MGr1-Ag/37LRP, is predictive of poor prognosis in gastric cancer. International journal of cancer. 2014; 135:2329–2337.

The list of primary antibodies used

Target	Usage	Source	Catalog number	Dilution
O-GlcNAc	WB	Abcam	Ab2739	1:1000
	IHC			1:200
p-ERK	WB	Cell signaling	8201	1:1000
	IHC			1:200
ERK 1/2	WB	Cell signaling	4695	1:1000
OGT	WB	Cell signaling	5368	1:1000
OGA	WB	Abcam	Ab124807	1:1000
CDK-2	WB	Millipore	2202539	1:1000
Cyclin D1	WB	Cell signaling	2978	1:1000
PCNA	WB	Cell signaling	13110	1:1000
β-actin	WB	Sigma-Aldrich	A2228	1:5000

The list and sequences of primers used for qRT-PCR

Primer	Sequence (5′–3′)
OGT-Forward	AGAAGGGCAGTGTTGCTGAAG
OGT-Reverse	TGATATTGGCTAGGTTATTCAGAGAGTCT
OGA-Forward	GCGGTGTGGTGGAAGGATT
OGA-Reverse	CCATTTCTGGAGCCTTCTAAAGAG

The list and sequences of primers used for RNA silence

Primer	Sequence (5′–3′)
OGT-si 1 Forward	CGCGUGCCAUCCAAAUUAAdTdT
OGT-si 1 Reverse	UUAAUUUGGAUGGCACGCGdTdT
OGT-si 2 Forward	GCACGGCUCUGAAACUUAAdTdT
OGT-si 2 Reverse	UUAAGUUUCAGAGCCGUGCdTdT
OGT-si 3 Forward	GGCAGAAGCUUAUUCGAAUdTdT
OGT-si 3 Reverse	AUUCGAAUAAUAAGCUUCUGCCdTdT

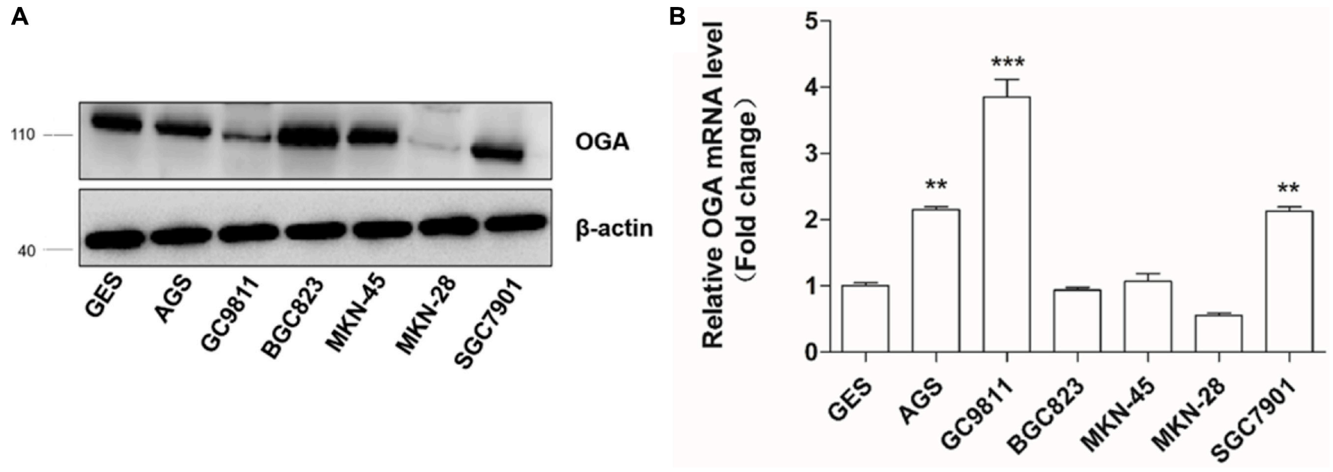

Supplementary Figure S1: OGA level in gastric cancer cells and normal gastric epithelial cells. (**A**) Western blot for OGA in six gastric cancer cell lines and normal gastric epithelial cells (GES). β-actin was a loading control. (**B**) Total RNA was harvested from six gastric cancer cell lines and GES cells, and levels of OGA mRNA were quantified by qRT–PCR and normalized to β-actin. Normalized OGA mRNA levels were presented relative to GES. Values represent mean ± SEM. * represents Student's *t*-test *$P < 0.05$, **$P < 0.01$ and ***$P < 0.001$.

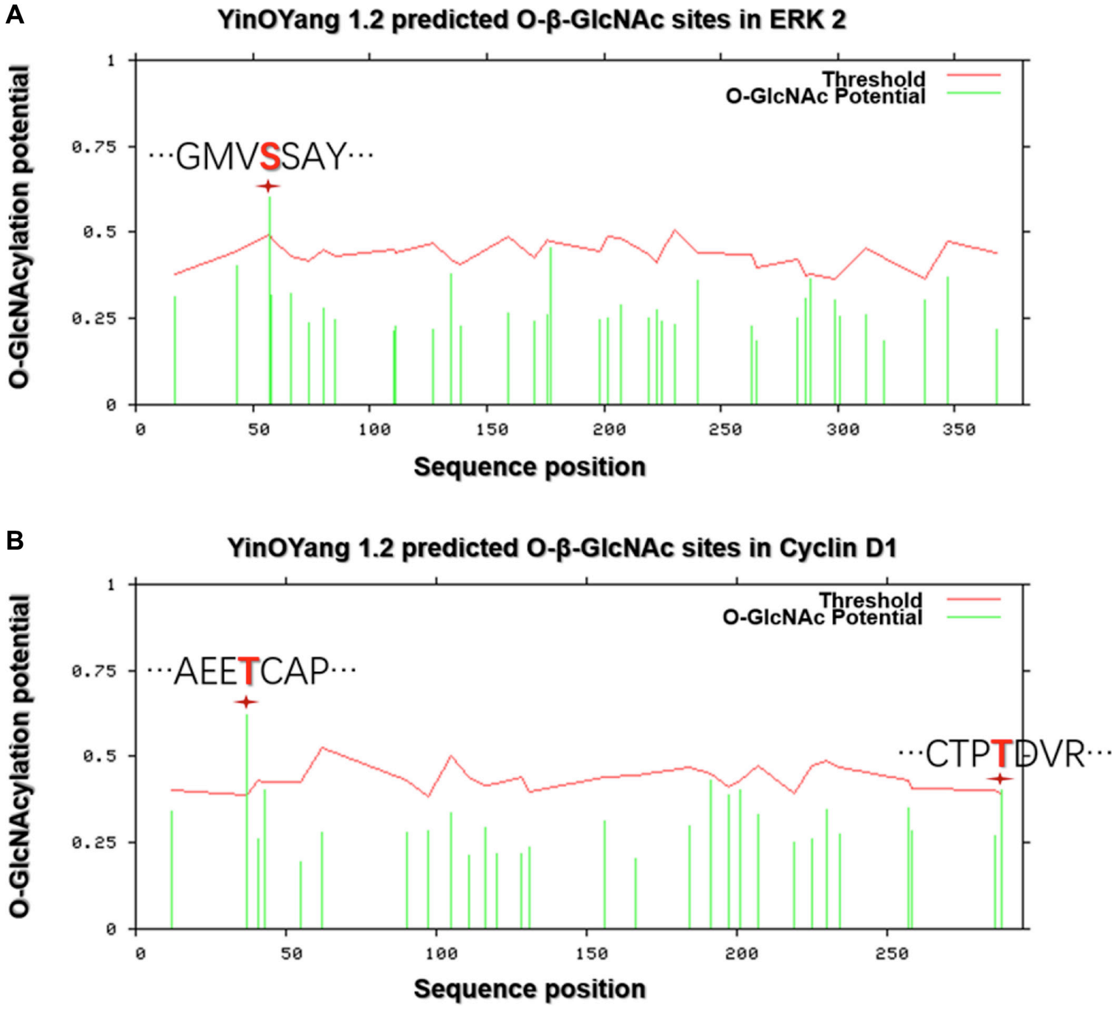

Supplementary Figure S2: Predicted potential sites for O-GlcNAc modification in human (A) ERK 2 and (B) cyclin D1. The positively predicted O-GlcNAc sites are shown with red asterisk at the top. The green vertical lines show the O-GlcNAc potential of Ser/Thr residue and the red horizontal wavy line indicates the threshold for modification potential.

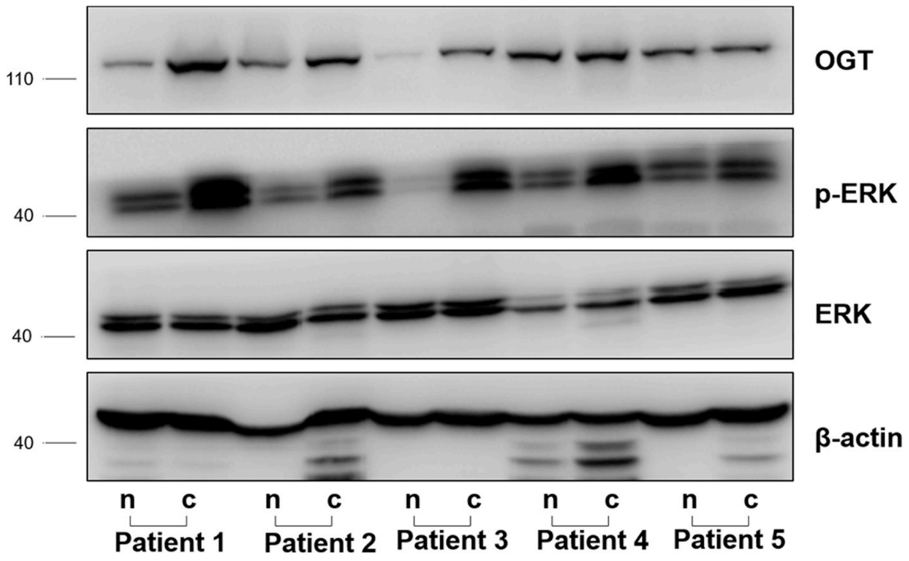

Supplementary Figure S3: The expression of OGT and ERK 1/2 in five cases of gastric cancerous tissues and matched normal counterparts. Western blot for OGT and ERK 1/2 in five gastric cancerous tissues and matched normal counterparts. β-actin was a loading control. “n” is shortened for “matched normal tissues” and “c” stands for “cancerous tissues”.
